# Anesthetic management of extracorporeal membrane oxygenation-supported aortic bypass surgery for atypical coarctation with severe left ventricular dysfunction: A case report

**DOI:** 10.1016/j.heliyon.2024.e35605

**Published:** 2024-08-02

**Authors:** Shusuke Okamoto, Takuya Okada, Norihiko Obata, Masahiko Iseki, Yu Yamane, Masaharu Nagae

**Affiliations:** a Department of Anesthesiology and Pain Clinic, Hyogo Prefectural Harima-Himeji General Medical Center, 3-264 Kamiya-cho, Himeji, 670-8560, Japan; b Division of Anesthesiology, Department of Surgery Related, Kobe University Graduate School of Medicine, 7-5-2 Kusunoki-cho, Chuo-ku, Kobe, 650-0017, Japan

**Keywords:** Atypical coarctation of aorta, Ascending-to-abdominal aorta bypass surgery, Extracorporeal membrane oxygenation, Severe left ventricular dysfunction, Case report

## Abstract

Atypical aortic coarctation is an exceedingly rare condition, and there are very few reported cases of anesthetic management for bypass surgery in patients with severe impaired cardiac function. We present the anesthetic management of a 68-year-old woman with atypical aortic coarctation due to Takayasu arteritis and severely impaired cardiac function, who underwent ascending-to-abdominal aorta bypass surgery under extracorporeal membrane oxygenation (ECMO).

The patient's severe cardiac dysfunction was due to sustained afterload from the coarctation, leading to recurrent episodes of heart failure. Surgical intervention was deemed necessary, and a decision was made to perform a bypass operation. The patient experienced a transient state of shock following induction of anesthesia, but subsequent perioperative care was safely managed with the implementation of ECMO. For bypass surgery performed on patients with severe cardiac dysfunction due to atypical coarctation of the aorta, it is crucial to prepare for potential circulatory collapse during anesthesia induction and the surgical procedure. This preparation includes meticulous planning of the anesthesia induction method and ensuring that ECMO can be established promptly if needed.

## Highlights

• Anesthetic strategy for aortic bypass in a patient with severe cardiac dysfunction.
• Preoperative ECMO standby for potential circulatory collapse.
• Anesthesia-induced tachycardia and hemodynamic instability.
• Successful surgery under ECMO, improving postoperative cardiac function.

## 1 Introduction

Atypical coarctation of the aorta, unlike its congenital counterpart that occurs in the aortic isthmus, denotes acquired stenosis that develops in a different segment of the aorta. The etiology varies, with a common association with acquired inflammation, particularly Takayasu arteritis [1,2]. Typically diagnosed in adolescence or adulthood, this condition presents as central hypertension proximal to the stenotic site and peripheral hypoperfusion, accompanied by symptoms such as headaches, intermittent claudication, and abdominal angina [3]. Prolonged progression and worsening stenosis can lead to complications, including aneurysms, heart failure, cerebrovascular events, and renal failure. The treatment options include surgical procedures (bypass surgery, artificial vascular replacement, patch insertion) and endovascular interventions, such as stent grafts. There are various choices for anastomosis sites when considering bypass surgery [2]. Atypical coarctation of the aorta is an exceedingly rare condition, accounting for 0.5–2% of all aortic coarctation cases [2]. While extracorporeal membrane oxygenation (ECMO) has been extensively reported for bypass surgery in cases of coarctation, there is a paucity of literature regarding the anesthetic management of bypass surgery in conditions characterized by severe cardiac dysfunction, as observed in the following [4,5].

Here, we present a case involving a patient with severe left ventricular dysfunction due to long-standing Takayasu arteritis-related stenosis of the descending aorta. The patient underwent ECMO-supported ascending-to-abdominal aorta bypass surgery. We provide insights into the anesthetic management of this challenging case.

## 2 Case presentation

A 68-year-old female patient (height: 166 cm, weight: 48.7 kg) had been diagnosed with atypical coarctation of the aorta and chronic heart failure due to Takayasu arteritis 15 years earlier. Pharmacological treatment with diuretics and beta-blockers was initiated. She had a pacemaker implanted 14 years ago for complete atrioventricular block, which was upgraded to a biventricular device (Cardiac Resynchronization Therapy Pacemaker: CRT-P) 9 years later due to declining cardiac function. However, her cardiac function gradually declined. In the past year, her heart function significantly deteriorated, leading to recurrent hospitalizations for heart failure. Further investigation revealed that a sustained afterload due to aortic coarctation was the primary cause of her worsening cardiac function. Additionally, unstable blood flow due to aortic coarctation was considered to have led to excessive secretion of renin, resulting in rapid fluctuations in afterload and repeated episodes of decompensated heart failure. Following discussions between the cardiology and cardiovascular surgery departments, surgical intervention was deemed necessary, and a decision was made to perform a bypass operation. Fluid simulations using preoperative computed tomography (CT) images were conducted to evaluate bypass options from the ascending aorta to the abdominal aorta, the axillary artery to the femoral artery, and immediately above and below the stenotic site. The results indicated that the bypass from the ascending aorta to the abdominal aorta would be the most advantageous for overall blood flow. Although transthoracic echocardiography (TTE) revealed severe mitral regurgitation, it was deemed that concurrent surgical intervention on the mitral valve would be excessively invasive. Therefore, only bypass surgery was undertaken.

**Fig. 1:**
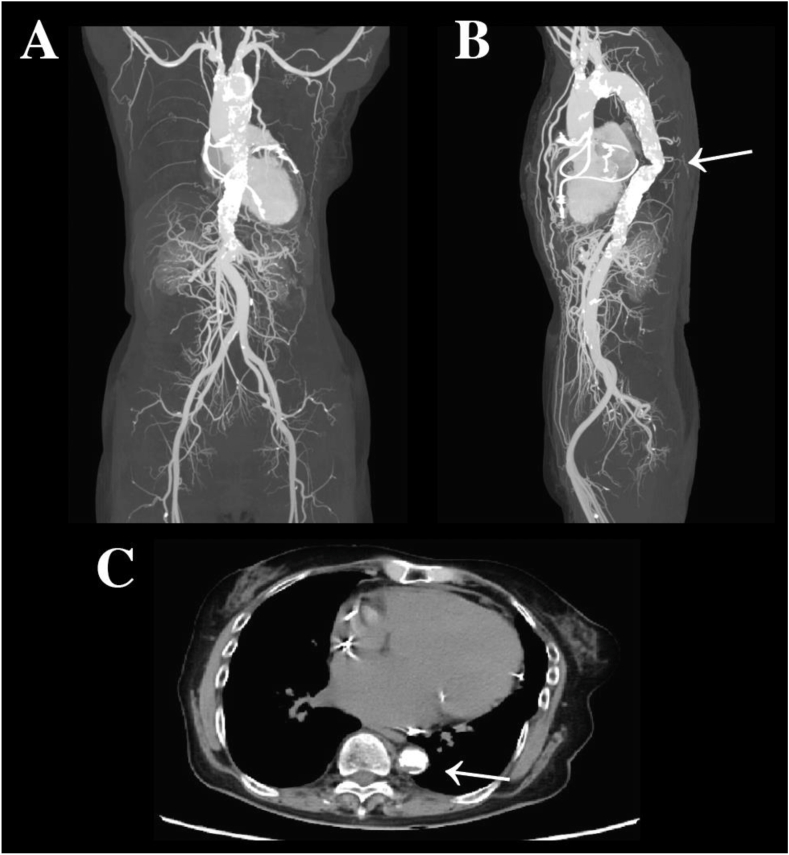
Preoperative computed tomography image of the aorta (A) Frontal view. (B) Left lateral view. (C) Axial plane. The region indicated by the arrow in (B) and (C) depicts the stenotic segment of the aorta characterized by advanced calcification.

The patient presented with a resting pulse rate of 64 beats per minute, blood pressure of 100/44 mmHg (upper limbs), percutaneous oxygen saturation of 98 % (Room Air), and ankle-brachial index (ABI) values of 0.63 on the right and 0.66 on the left, indicating moderate bilateral reduction in the value. She reported fatigue upon walking approximately 100 m, corresponding to New York Heart Association (NYHA) functional class II. The patient was prescribed oral medications, including losartan potassium 50 mg/day, carvedilol 20 mg/day, dapagliflozin propanediol hydrate 10 mg/day, and spironolactone 25 mg/day. Blood investigations showed a hemoglobin level of 11.5 g/dL, platelet count of 151,000/μL, blood urea nitrogen of 39 mg/dL, creatinine of 1.57 mg/dL, and NT-proBNP level of 22,626 pg/mL, indicating renal impairment and cardiac myocyte strain. TTE revealed a left ventricular ejection fraction of 20 % and widespread severely reduced wall motion. Doppler analysis demonstrated significant tricuspid regurgitation with a pressure gradient of 20.2 mmHg. Severe mitral regurgitation due to tethering and mild aortic regurgitation were also identified. Ventricular function was impaired bilaterally, with significant cardiac chamber enlargement and severe valve pathology. Chest and abdominal CT scans revealed highly calcified, narrowed segments in the descending thoracic aorta at the ninth to tenth thoracic vertebral levels, with the posterior lumen measuring approximately 3.8 mm × 11 mm (Fig. 1A, B and C). Marked dilation of the descending aorta and collateral circulation from the superior mesenteric artery were observed. Right renal artery stenosis was noted, with the Adamkiewicz artery originating between the 11th and 12th thoracic vertebrae. Arterial catheterization showed a significant pressure gradient at the stenotic lesion, with a maximum pressure difference of 65 mmHg. Right heart catheterization revealed a pulmonary artery wedge pressure of 11 mmHg, pulmonary artery pressure of 27/14 mmHg, right atrial pressure of 4 mmHg, cardiac output of 2.68 L/min, cardiac index of 1.72 L/min/m^2^, stroke volume of 43 mL, and stroke volume index of 27.6 mL/m^2^ (Fick's method). Although coronary angiography was not performed due to renal impairment, the adenosine stress testing showed no significant signs of ischemia or infarction. Carotid artery ultrasound revealed heterogeneous plaques and up to 50 % stenosis in both carotid arteries.

The anesthetic plan involved general anesthesia, and considering the significant cardiac dysfunction, arrangements were made to have extracorporeal membrane oxygenation (ECMO) on standby in the operating room to promptly address any circulatory collapse. Access vessels considered for ECMO initiation included the subclavian artery and femoral vein; however, the vessels were not secured before induction of anesthesia. The scheduled surgical procedure comprised a median sternotomy and midline laparotomy. The plan was to initially partially occlude the abdominal aorta and anastomose the synthetic grafts. Subsequently, the ascending aorta would be partially occluded for anastomosis, and the bypass graft would be positioned in front of the heart, passing through the diaphragm. In the event of circulatory collapse, particularly during partial occlusion of the ascending aorta, the prompt initiation of ECMO would have been performed. As with thoracoabdominal aortic aneurysm surgery, we paid close attention to spinal cord blood flow. Although temporary partial aortic clamping during anastomosis might have impacted spinal cord blood flow, the duration was brief. After consulting with the surgeon, we decided not to implement preemptive measures such as spinal drainage or the use of motor evoked potentials. Blood products prepared for transfusion included packed red blood cells, fresh frozen plasma, and concentrated platelet product. Continuous monitoring of bladder and rectal temperatures was planned for temperature management, with the utilization of a blanket-style warming device (Bair Hugger®) scheduled to commence prior to the initiation of surgery. Among the oral medications, only losartan potassium was discontinued on the day of surgery. Prophylactic antibiotic administration consisted of 1g of cefazolin and 1g of vancomycin prior to the commencement of surgery.

After entering the operating room, standard monitoring, as well as electroencephalogram (BIS®) and cerebral oximeter (INVOS®), were placed. Subsequently, an arterial pressure line was inserted into the left radial artery under local anesthesia. Induction of anesthesia was initiated with preoxygenation using oxygen at 6 L/min, followed by intravenous midazolam 3 mg, fentanyl 50 mcg, remifentanil at 0.05 mcg/kg/min, rocuronium 50 mg, and phenylephrine at 0.5 mcg/kg/min under invasive arterial pressure monitoring, followed by endotracheal intubation. Maintenance of anesthesia was achieved with 1 % sevoflurane and a continuous remifentanil infusion at 0.05 mcg/kg/min, along with phenylephrine at 0.5 mcg/kg/min for hemodynamic support. No significant fluctuations in vital signs were observed at the time of endotracheal intubation. Following endotracheal intubation, a transesophageal echocardiography (TEE) probe was placed. Additionally, to assess peripheral blood flow, an invasive arterial pressure line was inserted into the right dorsalis pedis artery. After approximately 8 minutes following endotracheal intubation, while preparing to place a pulmonary artery catheter, the heart rate suddenly increased from around 60 to 95 beats per minute, and systolic blood pressures dropped to the 60-mmHg range. Bolus phenylephrine administration temporarily increased the systolic blood pressure to approximately 75 mmHg. Subsequently, a sheath for the pulmonary artery catheter and the catheter itself (Swan-Ganz thermodilution catheter®) were promptly inserted. Despite additional bolus administration of phenylephrine, ephedrine, and norepinephrine, blood pressures gradually decreased. Anesthesia maintenance drugs were temporarily discontinued, and bolus doses of phenylephrine totaling 0.3mg, norepinephrine totaling 40 mcg, and adrenaline totaling 40 mcg were administered. However, the response was unfavorable, and systolic blood pressure dropped to 30 mmHg, necessitating the initiation of chest compressions. A 1 mg intravenous bolus of adrenaline and a 0.5 mg intravenous bolus of atropine were administered. Approximately 2 minutes later, the patient's blood pressure increased, and subsequently, infusions of dobutamine at 10 mcg/kg/min and norepinephrine at 0.18 mcg/kg/min were started. After stabilization of the patient's hemodynamics, anesthesia maintenance drugs were resumed. During the hypotensive episode following induction, PA catheter readings showed elevated PAP and CVP relative to systemic blood pressure. Furthermore, as TEE revealed no change in contractility, it is likely that right heart failure occurred. Although cardioversion was contemplated for tachycardia, it was postponed as hemodynamics stabilized with catecholamine administration.

**Fig. 2:**
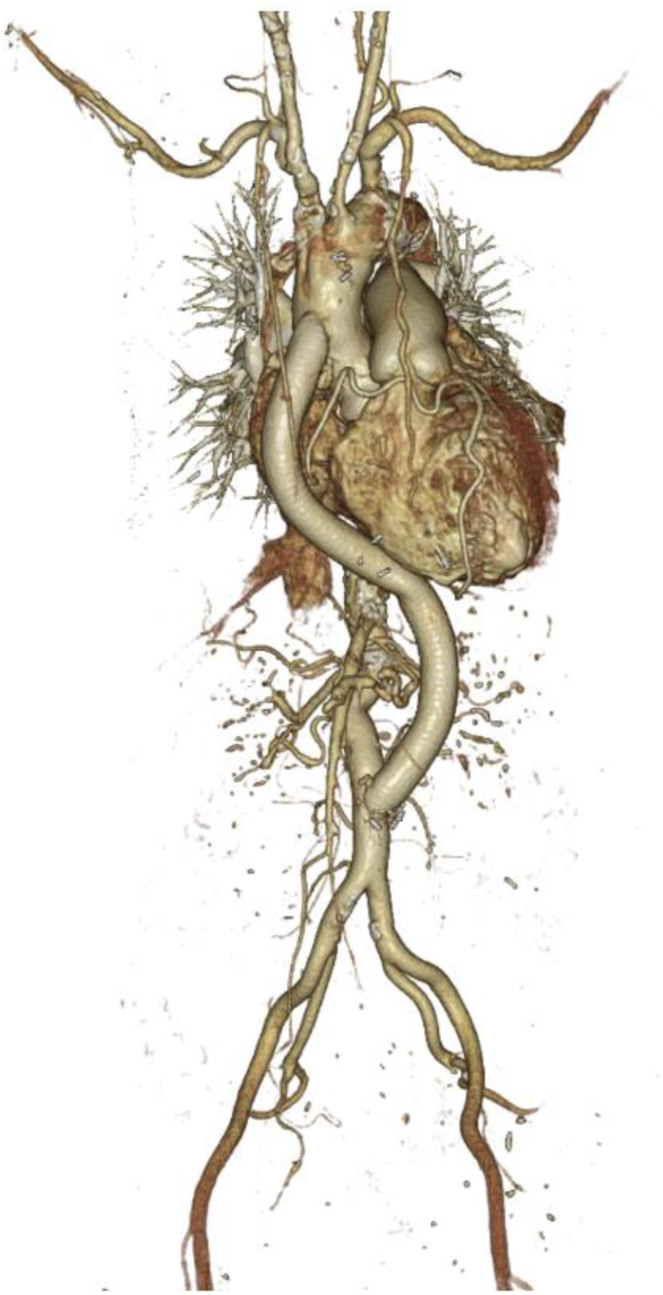
Postoperative 3D computed tomography images of the aorta.

**Fig. 3:**
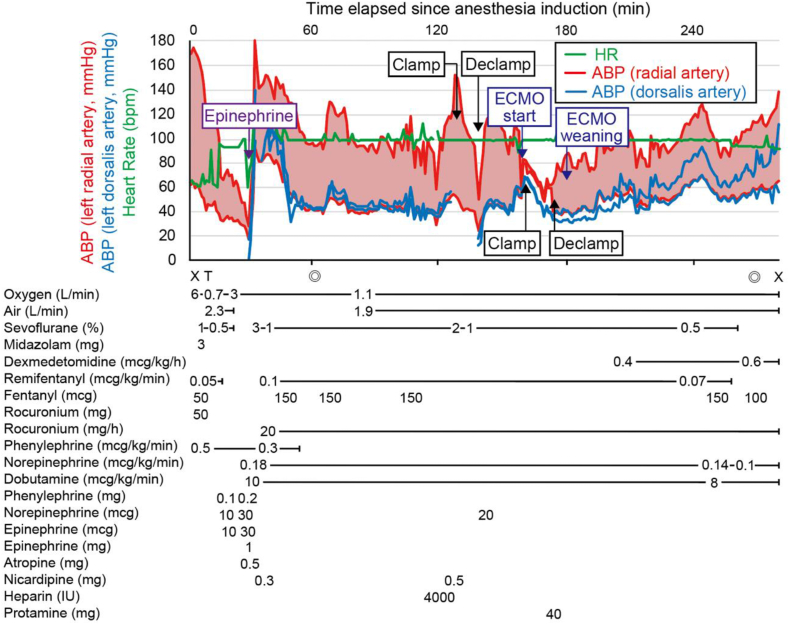
The patient's anesthesia record. X, start and end of anesthesia; ◎, start and end of surgery; T, intubation; HR, heart rate; ABP, arterial blood pressure.

Following consultation with the cardiovascular surgeon on the continuation of the surgery, it was determined to proceed, considering the anticipated deterioration in overall condition unless treatment for the underlying condition was performed. We also deliberated on minimizing the invasiveness of the surgery by considering a change in the bypass location. However, based on the results of preoperative fluid simulation, it was determined that the ascending aorta to abdominal aorta bypass would be preferable. Consequently, we decided to proceed with the procedure without altering the surgical approach. However, considering the patient's original cardiac function and the episode of severe hypotension requiring cardiopulmonary resuscitation after induction, the decision was made to perform ascending aorta anastomosis under ECMO support. The surgical procedure commenced with a median sternotomy and midline laparotomy. Tissues were dissected and exposed, and prior to aortic occlusion, a blood delivery cannula was placed in the aortic arch and a deairing cannula was placed in the right atrium, ensuring readiness for ECMO initiation at any time. Subsequently, anastomosis was performed on the abdominal aorta. Because of the pre-existing stenosis, sidebiting of the abdominal aorta was assessed to have minimal effects on hemodynamics. Hence, ECMO was not used for this part of the procedure, and the anastomosis was completed with a small amount of vasopressor cover. ECMO was initiated just before the partial occlusion of the ascending aorta, and during this phase, the pulse pressure nearly disappeared. However, circulation was maintained with ECMO assistance (flow rate of approximately 2.1–2.5 L/min), facilitating completion of the anastomosis. Subsequent declamping of the ascending aorta resulted in an increase in pulse pressure, confirming stable circulation. Thereafter, the ECMO flow rate was gradually reduced, and subsequently discontinued. The surgery concluded without significant complications (Fig. 2). The patient was transferred to the ICU while still intubated, and with ongoing continuous intravenous infusions of dexmedetomidine and fentanyl (Fig. 3). The intraoperative blood loss was 1532 mL, necessitating transfusion of 6 units (840 mL) of packed red blood cells, 8 units (960 mL) of fresh frozen plasma, and 10 units (200 mL) of platelet concentrate.

After admission to the ICU, the catecholamine doses were dobutamine 8 mcg/kg/min and norepinephrine 0.07 mcg/kg/min, which were subsequently successfully tapered. Postoperative recovery proceeded without complications, and extubation was achieved 18 h postoperatively. The pulmonary artery catheter and drains were removed on the first postoperative day. Catecholamine administration was discontinued on the second postoperative day, and the patient was transferred to the general ward. Due to persistent elevation of inflammatory makers and diagnosis of recurrent Takayasu arteritis on Positron Emission Tomography (PET)-CT, steroid therapy was initiated. Consequently, the hospitalization period was prolonged; however, gradual improvement in general condition and cardiac function led to discharge on postoperative day 36. After discharge, TTE demonstrated an enhanced left ventricular ejection fraction of 40 %, along with mild improvement in mitral regurgitation. Arterial catheterization revealed a diminished aortic pressure of 133/47 (72) mmHg and femoral artery pressure of 124/47 (72) mmHg, leading to a decreased pressure gradient of 9 mmHg. Furthermore, preoperative CT revealed stenosis at the base of the right renal artery; however, no further therapeutic intervention was pursued as the creatinine level improved from 1.57 mg/dL preoperatively to 1.00 mg/dL postoperatively. Additionally, upon discharge, the patient demonstrated independence in activities of daily living, remained asymptomatic at rest, and could ambulate short distances with a cane. The patient was classified as NYHA functional class II, with no significant change in functional status compared to preoperative levels. Nonetheless, there was an improvement in quality of life as the patient reported better sleep at night.

## 3 Discussion

Takayasu arteritis, an autoimmune condition, manifests as non-specific inflammation affecting elastic arteries, encompassing the aorta, major arterial branches, pulmonary arteries, and coronary arteries. The pathology results in arterial thickening, sclerosis, luminal constriction, dilation, and development of aneurysms. With regard to the anesthetic strategy, a meticulous preoperative assessment of the extent of vascular lesions in major organs, the degree of ischemic compromise, and potential complications is imperative. Tailored interventions might be required when complications such as cerebral infarction, disorders of cerebral circulation, aortic valve insufficiency, hypertension, and pulmonary hypertension coexist. Maintaining blood pressure to avoid hypotension during anesthesia is critical for sustaining adequate blood flow to vital organs [[6], [7], [8]]. In this case, along with the atypical coarctation, we identified right renal artery stenosis, and mild carotid artery stenosis. Due to the chronic progression, significant collateral circulation developed. However, the patient experienced recurrent episodes of abrupt blood pressure elevation, suggesting potential instability in distal blood flow from the stenotic site due to gradual decline in renal function and unstable renal blood flow.

There is still no consensus on the optimal blood pressure control in the anesthetic management of aortic coarctation, particularly regarding central and peripheral pressures relative to the stenotic site. However, due to the anatomical similarities with thoracoabdominal aortic aneurysm surgery, it is crucial to carefully manage blood pressure to avoid it becoming too low, thereby ensuring adequate spinal cord and renal protection. Some reports suggest maintaining a MAP of approximately 50 mmHg distal to the stenotic site to preserve spinal cord blood flow [9]. In this case, an invasive arterial pressure line was placed in the right dorsalis pedis artery after induction of anesthesia for the assessment of peripheral blood flow. However, as hemodynamics deteriorated and circulation was sustained with high doses of catecholamines, blood pressure in the lower limb arteries was slightly lower, averaging around 40–50 mmHg before and after the start of surgery. Following bypass grafting, lower limb arterial pressure gradually improved, with maintenance of an average blood pressure of 70 mmHg at the end of the surgery. Pulse pressures in the lower limbs also increased gradually, accompanied by an elevation in urine output. Although it was not considered in this case, the use of mannitol for renal protection might have been appropriate. Postoperatively, no evident lower limb paralysis or ischemic symptoms were observed.

There is a paucity of reports on the anesthetic management of atypical aortic coarctation with severely decreased cardiac function. While some previous reports focused on less invasive procedures, such as axillofemoral bypass, the degree of cardiac dysfunction in those reports was relatively mild compared to the challenging scenario presented in this case [10,11]. Our patient had a history of recurrent heart failure preoperatively, and her cardiac reserve was profoundly diminished. Although inotropic agents and oxygen were not initially required upon entering the operating room, induction of anesthesia triggered an increase in heart rate, leading to hemodynamic instability. During induction, phenylephrine was continuously administered to counteract the vasodilation induced by anesthetic agents. However, considering the compromised cardiac function in this patient, we should have considered using inotropic agents, such as epinephrine, norepinephrine, and dobutamine, before induction to prevent hypotension during induction. Additionally, we should have considered pre-placement the ECMO lines and carefully adjusting the ventilator settings by gradually increasing the inspiratory pressure of positive pressure ventilation. Nonetheless, it is crucial to remember that the use of inotropic agents increases myocardial oxygen demand and induces heightened myocardial calcium load, with the potential risks of arrhythmias, myocardial ischemia, and myocardial injury [12]. Although levosimendan is not approved for use in Japan, it may have been effective in this case. A small amount of midazolam was used at induction, which causes relatively mild circulatory depression. However, consideration should also be given to the use of ketamine as an alternative. Additionally, while not approved for use in Japan, etomidate and similar anesthetics are also considered suitable for minimizing cardiac depression.

Intraoperative management of patients with low cardiac function, as in this case, greatly benefits from the use of TEE and PA catheters for hemodynamic monitoring [4,13]. Particularly in scenarios where ECMO may be utilized, these monitoring tools can significantly enhance intraoperative and postoperative management. Therefore, their early implementation should be considered. Moving forward, it is essential to further investigate the optimal use of these monitoring tools to improve patient care.

In the event of circulatory collapse necessitating cardiopulmonary resuscitation (CPR) during anesthesia, if standard resuscitation measures, such as adrenaline administration, prove ineffective, consideration should be given to extracorporeal cardio-pulmonary resuscitation (ECPR). The femoral artery approach is the primary choice for vascular access in ECPR [14]. However, in cases of aortic coarctation, femoral artery access might not efficiently deliver an adequate blood supply to the upper body, making alternatives, such as the subclavian, axillary, or innominate arteries, reasonable options. Nevertheless, these alternatives pose a higher rate of puncture difficulty compared to the femoral artery, and might necessitate interventions such as cut-down procedures in certain cases. In our case, the subclavian artery was considered as an emergency approach, but was not actually secured. Considering the potential urgency, it might have been prudent to secure the subclavian artery preoperatively. In surgeries involving anatomical regions similar to this case, such as thoracoabdominal aortic aneurysm surgery, not only veno-arterial (VA)-ECMO but also methods like Left Ventricular (LV) bypass and Cardiopulmonary bypass (CPB) have been reported for extracorporeal circulation [[15], [16], [17]]. Anesthesiologists must be knowledgeable about the characteristics, access vessels, advantages, disadvantages, and activated clotting time (ACT) related to each method (Table 1, Table 2) [16,[18], [19], [20], [21]].

**Table 1:** Comparison of central and peripheral circulatory support.

	Central support	Peripheral support
Advantages	・ Antegrade flow ・ Faster when the chest is open	・ Less invasive (Not require open chest) ・ Faster when the chest is not open
Disadvantages	・ More invasive (Need open chest, Higher risk of bleeding)	・ Dual circulation (North-South or Harlequin syndrome) ・ Increases the afterload on the left ventricle ・ Vascular complications (Limb ischemia)

**Table 2:** Comparison of VA-ECMO, LV bypass and CPB.

	VA - ECMO		LV bypass		CPB
Access	RA-Ao (central)	RA-FA (peripheral)	LA-Ao (central)	LA-FA (peripheral)	RA-FA/Ao
Advantages	Maintain oxygenation is available Advantageous in the presence of right heart failure		More physiological pulmonary circulation		Cardiotomy suction is available
	Maintain upper body perfusion	Maintain lower body perfusion	Maintain upper body perfusion	Maintain lower body perfusion	
Disadvantages			Unable to maintain oxygenation when the lung function declines		Require systemic heparinization Increase bleeding/Increase inflammatory response
	Low operability of perfusion in the lower body, including renal and Adamkiewicz artery	Risk of upper body hypoperfusion in the event of an accidental drop in cardiac output	Low operability of perfusion in the lower body, including renal and Adamkiewicz artery	Risk of upper body hypoperfusion in the event of an accidental drop in cardiac output	
Target ACT time	180–220 s	180–220 s	> 200 s	> 200 s	> 480 s

RA, right atrium; LA, left atrium; Ao, aorta; FA, femoral artery; ACT, active clotting time.

Regarding the surgical procedure, the stenosis in this case was located above the renal arteries at the tenth thoracic vertebra level. However, to facilitate anastomosis, the graft was connected below the renal arteries. During the surgery, both the ascending aorta and the abdominal aorta were clamped. When the ascending aorta was clamped, blood from the heart temporarily could not flow downstream, leading to a reduction in the blood returning to the left ventricle, thereby decreasing preload. Additionally, this sharply increased the resistance (afterload) against which the heart pumps blood. Renal blood flow was also temporarily reduced due to a decrease in cardiac output. Clamping the abdominal aorta temporarily blocked blood flow to the lower body, potentially increasing venous return and preload. Since there is minimal direct impairment to the blood flow to the upper body (including the heart), a significant increase in afterload might be avoided. However, the increase in systemic blood pressure due to blocked blood flow to the lower body could indirectly raise afterload. As the renal arteries branch off from the abdominal aorta, clamping the abdominal aorta directly reduces renal blood flow, decreasing oxygen supply to the kidneys and posing a risk of renal dysfunction. It is essential to be aware that prolonged clamping can cause acute kidney injury.

Typically, postoperative adverse prognostic factors for aortic coarctation include disease duration, advanced age, complications such as renal artery stenosis, and the presence of concomitant aortic valve insufficiency [22]. In this case, there was a considerably long duration of 15 years from diagnosis, and although it was unilateral, renal artery stenosis was observed. However, postoperatively, despite the recurrence of Takayasu arteritis and episodes of supraventricular tachyarrhythmia, blood pressure control was satisfactory. Transthoracic echocardiogram at the 4-month postoperative mark showed improvement of left ventricular ejection fraction to 40 %, with no recurrence of heart failure, indicating a favorable outcome.

## 4 Conclusion

We presented the anesthetic management for ascending aorta to abdominal aorta bypass surgery in a patient with atypical aortic coarctation and severely impaired cardiac function. The patient experienced a transient state of shock following induction of anesthesia, but subsequent perioperative care was safely managed with the implementation of ECMO. For bypass surgery performed on patients with severe cardiac dysfunction due to atypical coarctation of the aorta, it is crucial to prepare for potential circulatory collapse during anesthesia induction and the surgical procedure. This preparation includes meticulous planning of the anesthesia induction method and ensuring that ECMO can be established promptly if needed.

## Informed consent

Written informed consent was obtained from the patient for publication of this case report.

## Funding statement

This work did not receive any specific grant from funding agencies in the public, commercial, or not-for-profit sectors.

## Data availability statement

Data included in article/supp. material/referenced in article.

## CRediT authorship contribution statement

**Shusuke Okamoto:** Writing – original draft, Data curation. **Takuya Okada:** Writing – original draft, Data curation. **Norihiko Obata:** Writing – review & editing, Supervision. **Masahiko Iseki:** Writing – original draft, Data curation. **Yu Yamane:** Writing – original draft, Data curation. **Masaharu Nagae:** Writing – review & editing, Supervision.

## Declaration of competing interest

The authors declare that they have no known competing financial interests or personal relationships that could have appeared to influence the work reported in this paper.
